# Updated Evaluation of Robotic- and Video-Assisted Thoracoscopic Lobectomy or Segmentectomy for Lung Cancer: A Systematic Review and Meta-Analysis

**DOI:** 10.3389/fonc.2022.853530

**Published:** 2022-04-12

**Authors:** Jianyong Zhang, Qingbo Feng, Yanruo Huang, Lanwei Ouyang, Fengming Luo

**Affiliations:** ^1^ Regenerative Medicine Research Center, West China Hospital, Sichuan University, Chengdu, China; ^2^ Department of Liver Surgery and Liver Transplantation Centre, West China Hospital, Sichuan University, Chengdu, China; ^3^ Department of Anesthesiology, The Affiliated Hospital of Guizhou Medical University, Guizhou, China; ^4^ Department of Thoracic Surgery, The 3rd Affiliated Hospital of Chengdu Medical College, Pidu District People’s Hospital, Chengdu, China; ^5^ Department of Respiratory and Critical Care Medicine, West China Hospital, Sichuan University, Chengdu, China

**Keywords:** lung cancer, video-assisted thoracic surgery, robot-assisted thoracic surgery, meta-analysis, systematic review

## Abstract

**Objectives:**

Robot-assisted thoracic surgery (RATS) and video-assisted thoracic surgery (VATS) are the two principal minimally invasive surgical approaches for patients with lung cancer. This study aimed at comparing the long-term and short-term outcomes of RATS and VATS for lung cancer.

**Methods:**

A comprehensive search for studies that compared RATS versus VATS for lung cancer published until November 31, 2021, was conducted. Data on perioperative outcomes and oncologic outcomes were subjected to meta-analysis. PubMed, Web of Science, and EMBASE were searched based on a defined search strategy to identify eligible studies before November 2021.

**Results:**

Twenty-six studies comparing 45,733 patients (14,271 and 31,462 patients who underwent RATS and VATS, respectively) were included. The present meta-analysis showed that there were no significant differences in operative time, any complications, tumor size, chest drain duration, R0 resection rate, lymph station, 5-year overall survival, and recurrence rate. However, compared with the VATS group, the RATS group had less blood loss, a lower conversion rate to open, a shorter length of hospital stay, more lymph node dissection, and better 5-year disease-free survival.

**Conclusions:**

RATS is a safe and feasible alternative to VATS for patients with lung cancer.

## Introduction

Lung cancer is still the most common malignancy worldwide (1, 2), and the main effective treatment is surgery for early-stage non-small-cell lung cancer (NSCLC) (3, 4). Emerging evidence has revealed that video-assisted thoracic surgery (VATS) is also a safe and effective treatment method for NSCLC compared with conventional thoracotomy (5–7). Additionally, it has been reported that VATS patients have less postoperative pain, shorter hospital length of stay, fewer complications, faster physical recovery, and better postoperative lung function outcomes than thoracotomy patients (5, 6, 8). Based on these advantages, VATS (including uniportal, two-port, three-port, or four-port) has been widely used to treat lung cancer. Although VATS is widely used in thoracic surgery based on its advantages, it also suffers from shortcomings, such as two-dimensional vision, difficult hand–eye coordination, amplification of hand tremor, steep learning curve, lack of flexibility, and limited ranges of instrument movement (8). Based on new emerging technologies and advances in medical knowledge, robot-assisted thoracic surgery (RATS) may be an alternative to VATS, as RATS can provide 3D high-definition visualization over the operative field, increase comfort for the surgeon, and improve the precision of manipulations with tremor filtration and instrument dexterity (9). Some studies have confirmed the safety and feasibility of RATS (7, 9) and emphasized its advantages in less bleeding, lower conversion rate, shorter hospital stay, more harvested lymph nodes and stations, lower overall complication rate, and lower recurrence rate (8) due to 3D high-definition visualization, tremor filtration, and instrument dexterity of robotic surgery systems. With several new studies about RATS and VATS for NSCLC published recently, a new updated meta-analysis is urgently needed to compare the perioperative and oncologic outcomes of RATS and VATS for NSCLC.

## Methods

### Data Sources and Search Strategy

This study was registered at PROSPERO under registration number CRD42021298987 and reported on the basis of the PRISMA guidelines (10). Studies investigating RATS versus VATS for NSCLC were systematically searched in PubMed, Web of Science, and EMBASE before November 31, 2021, by two independent investigators (JZ and QF). The search terms used were (“robotic surgery” OR “robot-assisted” OR robot OR robotic OR RATS) AND (“video-assisted surgery” OR “video-assisted” OR video OR thoracoscopic OR VATS) AND (“lung neoplasms” OR “lung cancer” OR “non-small-cell lung cancer” or NSCLC) and (“lung resection” OR “pulmonary resection” OR lobectomy OR segmentectomy), either individually or in combination. The “related articles” function was used to broaden the search, and all citations were considered for relevance. A manual search of the references of publication was adopted to prevent missing relevant studies.

### Inclusion and Exclusion Criteria

Two investigators (JZ and QF) independently reviewed the currently available literature, screened all titles and abstracts, and identified eligible studies according to the following criteria.

The inclusion criteria were as follows (1): participants: all patients had lung cancer defined histologically (2); types of interventions: RATS and VATS (3); study type: randomized controlled trials (RCTs), propensity score matching studies, retrospective studies, cohort studies, and case–control studies comparing RATS and VATS with NSCLC patients; if repeated studies were published from the same center, we captured the latest data and PSM data for analysis (4); at least one outcome was reported in the literature, including operation time, intraoperative bleeding, tumor size, R0 rate, conversion rate, lymph node harvested, and spleen preservation rate; and (5) language restrictions: English.

The exclusion criteria were as follows (1): conference abstracts, editorials, letters, and case reports and (2) no comparative analysis between RATS and VATS.

### Data Extraction and Quality Assessment

The original data from all candidate articles were independently assessed and extracted by two reviewers (JZ and QF) by using a unified datasheet, and any ambiguity was resolved by a third researcher (YH). The major data extraction includes the following: name of first author, publication year, study design, country, number of patients, mean age, sex, operative times, tumor size, bleeding, hospitalization, overall complication, overall complications, mortality, blood transfusion, and R0 rate. The quality of the eligible studies was assessed by the Newcastle–Ottawa Scale (NOS) by two different assessors (11). Every included study was independently evaluated by two authors (JZ and YH), and an NOS score>6 was considered high quality.

### Statistical Analysis

Review Manager 5.3 software was used for statistical analyses. The 95% confidence interval (CI) and mean difference (MD) were used for continuous data, while categorical variables were used as odds ratios (ORs). The method originally described by Hozo et al. was used to convert medians with ranges into means with standard deviations (12). Potential publication bias was visually assessed by Begg’s funnel plot and Egger’s test. Statistical heterogeneity was quantified using the I^2^ value. A fixed-effect model (FEM) was adopted when heterogeneity was low or moderate (I^2^ <50%), while heterogeneity was high (I^2^ ≥50%). A random-effect model (REM) was used.

## Results

### Characteristics of the Included Studies

**Figure 1:**
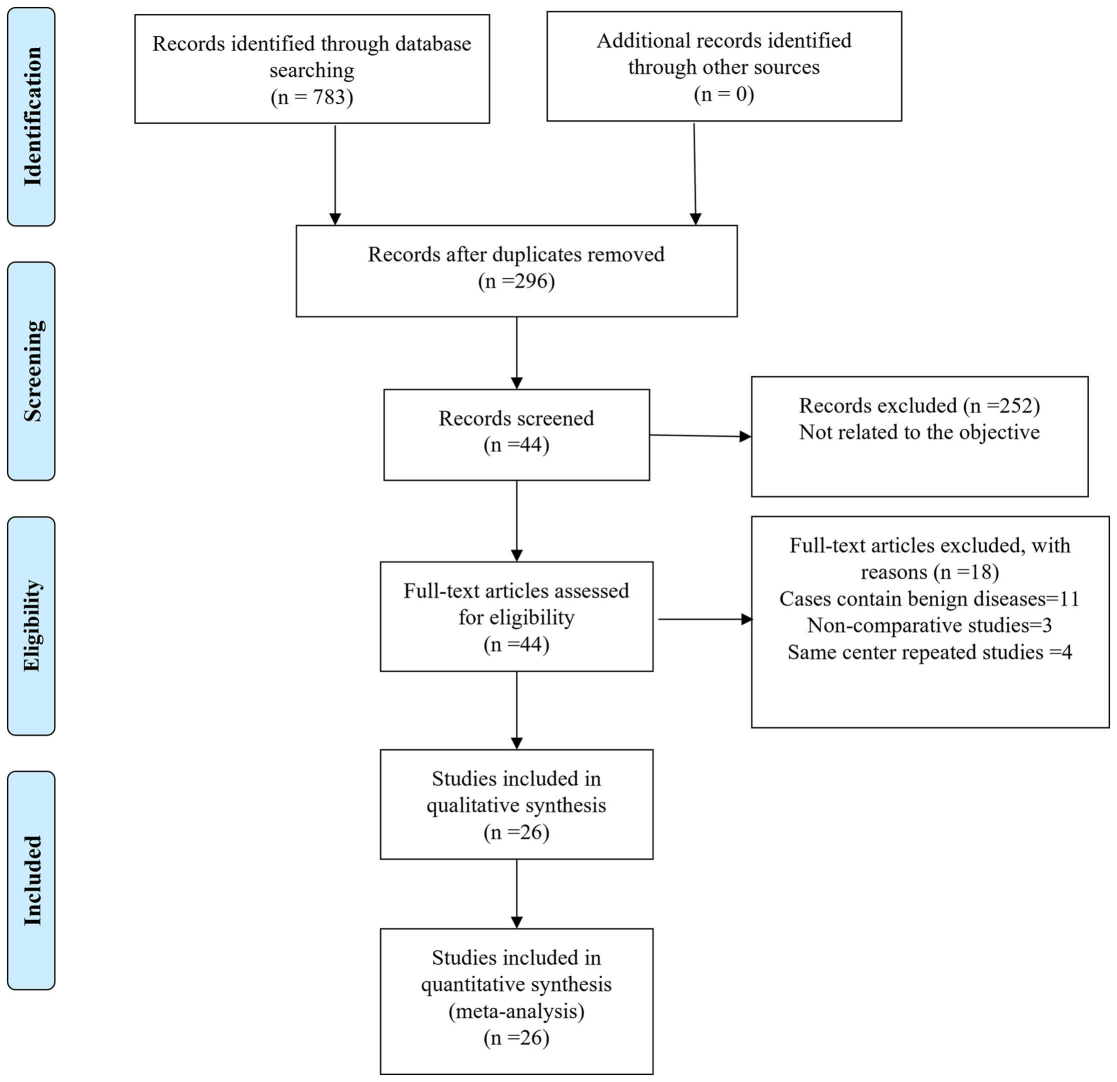
Flowchart of study identification and selection.

Finally, a total of 783 relevant English publications from the various electronic databases were identified. Finally, according to the inclusion criteria, 26 studies (13–38) comparing RATS and VATS in a total of 45,733 patients (14,271 and 31,462 underwent RATS and VATS, respectively) were included for further analysis. A flow diagram of our analysis protocol is shown in **Figure 1**. The general information and summary of NOS scores of all the included studies are given in **Table 1**.

**Table 1:** The main characteristics and NOS of the included studies.

First author, year	Country	Type	Period	Arms	NP(number of patients)	Number of segmentectomies	Age, median, y	Male/female	BMI (kg/m^2^)	Newcastle–Ottawa Scale (score)	Outcome
Jang 2011 (13)	Korea	RS	2006–2009	RATS	40	NA	64.2 ± 9.9	23/17	NA	7	(1) (2) (3) (4) (5) (7) (9) (10)
				VATS	40	NA	59.6 ± 10.1	24/16	NA		
Deen 2014 (14)	USA	RS	2008–2012	RATS	57	NA	68	19/38	NA	7	(1) (4) (7)
				VATS	58	NA	65	21/37	NA		
Lee 2015 (15)	USA	RS	2009–2014	RATS	53	NA	71 (52–85)	30/23	26.5 (20–43)	8	(1) (5) (7) (9)
				VATS	158	NA	72 (43–88)	56/102	26.3 (17–47)		
Mungo 2016 (16)	USA	RS	2007–2014	RATS	53	6	66 (60–71)	NA	26.7 (23.1–30.9)	7	(3) (4) (6) (7) (9) (13)
				VATS	80	1	67.5 (62-74)	NA	26.0 (24.2–28.1)		
Bao 2016 (17)	China	PSM	2014–2015	RATS	69	7	58.6 ± 8.8	26/43	NA	7	(1) (2) (4) (5) (6) (7) (9) (10)
				VATS	69	7	59.9 ± 9.7	22/47	NA		
Jeffrey 2016 (18)	USA	PSM	2010–2012	RATS	1,938	NA	68 (61–74)	839/1099	NA	9	(3) (5) (7) (8)
				VATS	1,938	NA	69 (62–74)	859/1079	NA		
Yang 2017 (19)	USA	RS, PSM	2002–2012	RATS	172	NA	68.0 (10.2)	98/74	NA	8	(3) (4) (7) (8) (9) (10) (11) (12) (13)
				VATS	141	NA	67.5 (10.0)	88/53	NA		
Li 2019 (20)	China	RS	2014–2017	RATS	36	NA	57.2 ± 8.9	17/19	NA	8	(1) (3) (5) (6) (7) (8) (9) (10) (13)
				VATS	85	NA	59.7 ± 8.8	38/47	NA		
Merritt 2019 (21)	USA	RS	2014–2018	RATS	114	NA	64.82 ± 11.35	46/68	NA	7	(1) (3) (4) (5) (7) (8) (9)
				VATS	114	NA	62.52 ± 10.65	49/65	NA		
LiJT 2019 (22)	China	RS	2013–2016	RATS	230	NA	55.6 ± 10.2	76/154	NA	7	(1) (3) (4) (5) (6) (7) (9) (10)
				VATS	230	NA	56.0 ± 9.7	80/150	NA		
Nelson 2019 (23)	USA	RS	2011–2017	RATS	106	NA	67 ± 10	NA	NA	8	(1) (2) (3) (7) (8)
				VATS	301	NA	66 ± 9	NA	NA		
Huang 2019 (24)	USA	RS	2010–2015	RATS	61	4	67 (31–85)	27/34	NA	7	(3) (4) (6) (7) (9)
				VATS	105	4	67 (40–91)	58/47	NA		
Hennon 2019 (25)	USA	RS	2010–2014	RATS	5,470	NA	66.8 (9.8)	2,421/3049	NA	7	(3) (7) (9) (11)
				VATS	17,545	NA	66.6 (10.2)	7,696/9849	NA		
Veluswamy 2020 (26)	USA	RS	2008–2013	RATS	338	NA	73.0 (8.0)	148/190	NA	9	(1) (4) (6)
				VATS	1,230	NA	72.0 (7.0)	542/688	NA		
Qiu 2020 (27)	China	RS	2012–2017	RATS	49	NA	NA	NA	NA	7	(1) (2) (6) (7) (9)
				VATS	73	NA	NA	NA	NA		
Zhou 2020 (28)	China	RS	2011–2018	RATS	50	50	54.7 ± 10.3	15/35	23.7 ± 3.6	9	(1) (2) (4) (5) (6) (7) (9) (11) (12) (13)
				VATS	80	80	57.7 ± 9.7	26/54	23.7 ± 2.8		
Haruki 2020 (29)	Japan	RS, PSM	2011–2018	RATS	49	NA	70 ± 12	24/28	23.8 ± 3.5	8	(1) (2) (4) (5) (6) (12) (13)
				VATS	49	NA	68 ± 11	24/25	23.5 ± 4.6		
Zhang 2020 (30)	China	RS	2015–2019	RATS	257	257	53.53 ± 10.96	84/173	23.13 ± 2.71	7	(1) (3) (4) (5) (6) (7) (8)
				VATS	257	257	52.21 ± 11.89	89/168	23.02 ± 3.88		
Sesti 2020 (31)	USA	RS, PSM	2008–2013	RATS	409	NA	73 (65–91)	188/221	NA	8	(5) (7)
				VATS	409	NA	74 (65–88)	176/233	NA		
Williams 2020 (32)	USA	RS	2014–2018	RATS	80	NA	66 ± 1.2	37/43	28.3 ± 0.8	8	(1) (2) (4) (7) (9)
				VATS	139	NA	66.7 ± 0.85	66/73	29.3 ± 0.8		
Kent 2021 (33)	USA	RS, PSM	2013–2019	RATS	1,711	NA	67.8 ± 9.7	742/969	28.0 ± 6.2	8	(2) (3) (4) (5) (6) (7)
				VATS	1,711	NA	67.9 ± 9.0	759/952	27.7 ± 5.9		
Jin 2021 (34)	China	RCT	2017–2020	RATS	157	NA	61 (54–66)	81/76	23.4 (21.7–25.6)	9	(1) (2) (4) (6) (7) (9) (10)
				VATS	163	NA	62 (53–68)	76/87	22.9 (21.4–24.4)		
Gallina 2021 (35)	ITALY	RS	2010–2019	RATS	237	NA	65.2 ± 6.3	134/103	NA	8	(4) (5) (7) (9)
				VATS	110	NA	64 ± 5.3	63/47	NA		
Seder 2021 (36)	USA	RS, PSM	2015–2019	RATS	2,133	252	66.62 (9.1)	911/1222	34.9 (4.6)	8	(1) (3) (4) (7)
				VATS	5,974	631	66.65 (8.9)	2,598/3377	34.6 (4.5)		
Chen 2021 (37)	China	RS, PSM	2016–2018	RATS	364	NA	59.3 ± 10.2	190/174	23.3 ± 2.9	8	(4) (5) (7) (9)
				VATS	364	NA	59.0 ± 10.1	190/174	23.3 ± 3.0		
Veronesi 2021 (38)	ITALY	RCT	2017–2018	RATS	38	NA	69 ± 8.3	21/17	27 ± 4.0	8	(3) (4) (5) (7) (8) (10)
				VATS	39	NA	69 ± 7.3	23/16	26 ± 4.1		

RATS, robot-assisted thoracic surgery; VATS, video-assisted thoracic surgery; NOS, Newcastle–Ottawa Scale; NA, not applicable; RCT, randomized controlled trial; PSM, propensity score matching study; RS, retrospective study.

Outcomes: (1) operative time, (2) blood loss, (3) conversion to open surgery, (4) any complications, (5) tumor size, (6) chest drain duration, (7) length of hospital stay, (8) R0 resection rate, (9) lymph node dissection, (10) lymph node station, (11) 5-year overall survival, (12) 5-year disease-free survival, (13) recurrence rate.

### Intraoperative Outcomes

**Figure 2:**
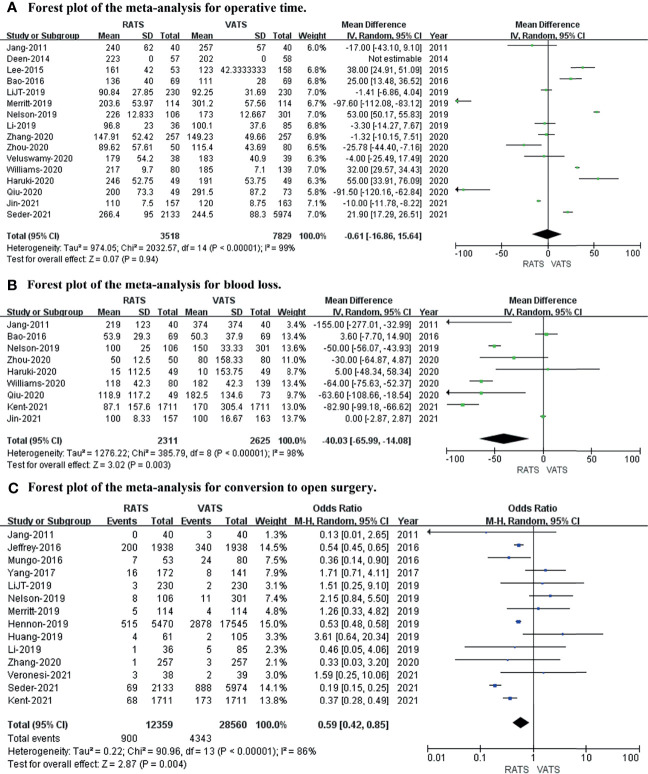
Forest plot of the meta-analysis for intraoperative outcomes. **(A)** Forest plot of the meta-analysis for operative time. **(B)** Forest plot of the meta-analysis for blood loss. **(C)** Forest plot of the meta-analysis for conversion to open surgery.

To evaluate the intraoperative outcomes, we compared the operative time, blood loss, and conversion to open surgery in patients who underwent RATS and VATS. The heterogeneity of the operative time, blood loss, and conversion to open surgery was extremely high (I^2^ = 99%, 98%, 86%, respectively). Sixteen studies that encompassed 11,347 patients (3,518 and 7,859 underwent RATS and VATS, respectively) reported operative times. The meta-analysis showed no difference in operative time between the two groups (*p* value = 0.94; 95% CI -16.86 to 15.64). Nine studies recorded intraoperative blood loss, and fourteen studies provided data on conversion to open surgery. A meta-analysis of these data suggested that RATS was associated with less bleeding (*p* value = 0.003; 95% CI -65.99 to -14.08) and a lower conversion rate to open surgery (*p* value = 0.004; 95% CI 0.45 to 0.85) than VATS (shown in **Figure 2**).

### Postoperative Outcomes

**Figure 3:**
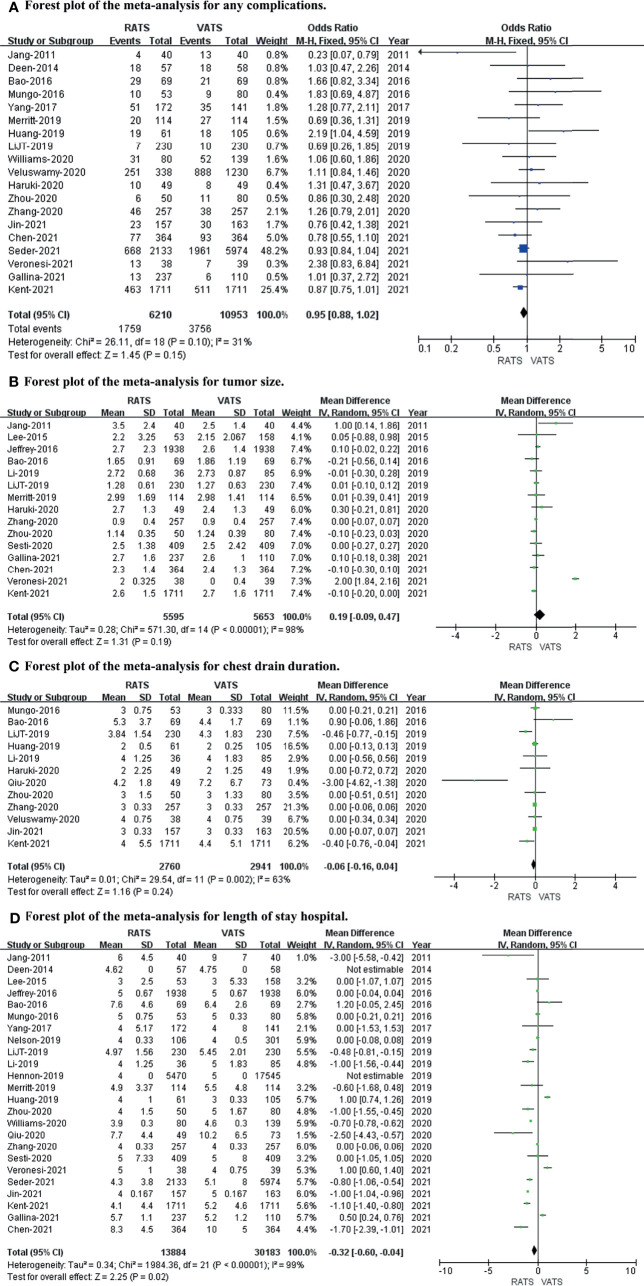
Forest plot of the meta-analysis for postoperative outcomes. **(A)** Forest plot of the meta-analysis for any complications. **(B)** Forest plot of the meta-analysis for tumor size. **(C)** Forest plot of the meta-analysis for chest drain duration. **(D)** Forest plot of the meta-analysis for length of hospital stay.

We used any complications, tumor size, chest drain duration, and length of hospital stay to evaluate the postoperative outcome. After meta-analysis, the length of hospital stay (*p* value = 0.02; 95% CI -0.60 to -0.04; I^2^ = 99%) was slightly longer in the VATS group than in the RATS group, with no differences in any complications (*p* value = 0.15; 95% CI 0.88 to 1.02; I^2^ = 31%), tumor size (*p* value = 0.19; 95% CI -0.09 to 0.47; I^2^ = 98%), or chest drain duration (*p* value = 0.24; 95% CI -0.16 to 0.04; I^2^ = 63%) between the two approaches (shown in **Figure 3**).

### Short−Term Oncological Outcomes

**Figure 4:**
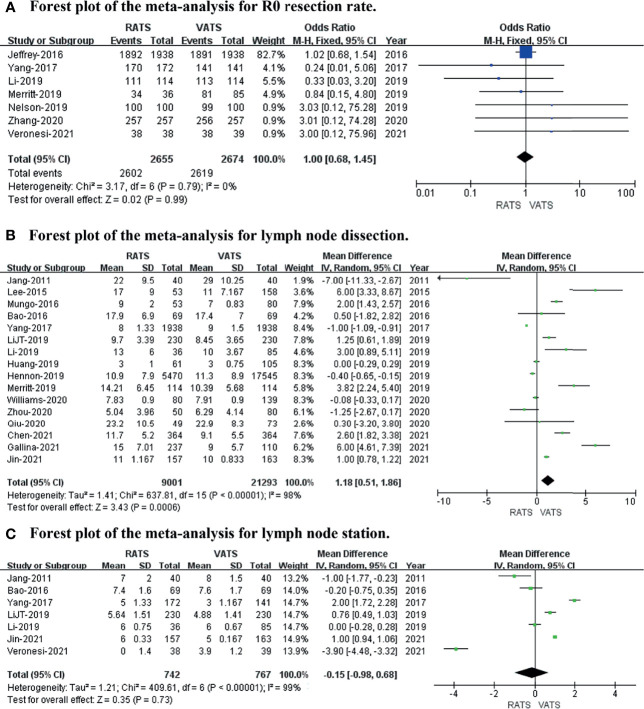
Forest plot of the meta-analysis for short−term oncological outcomes. **(A)** Forest plot of the meta-analysis for the R0 resection rate. **(B)** Forest plot of the meta-analysis for lymph node dissection. **(C)** Forest plot of the meta-analysis for lymph stations.

To evaluate short-term oncological outcomes, the R0 resection rate, lymph node dissection, and lymph stations were included in the meta-analysis. The results revealed no difference in R0 resection rate (*p* value = 0.99; 95% CI 0.68 to 1.45; I^2^ = 0%) or lymph station (*p* value = 0.73; 95% CI -0.98 to 0.68; I^2^ = 99%), while the lymph node dissection (*p* value = 0.0006; 95% CI 0.51 to 1.86; I^2^ = 98%) was captured in RATS more than in VATS (shown in **Figure 4**).

### Long−Term Oncological Outcomes

**Figure 5:**
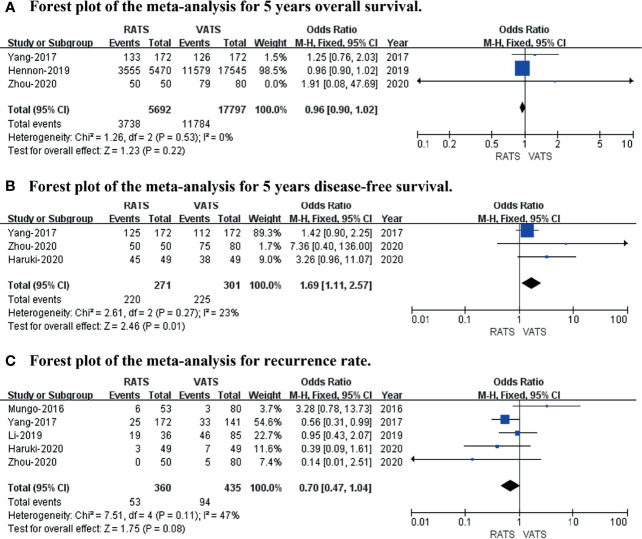
Forest plot of the meta-analysis for long−term oncological outcomes. **(A)** Forest plot of the meta-analysis for 5-year overall survival. **(B)** Forest plot of the meta-analysis for 5-year disease-free survival. **(C)** Forest plot of the meta-analysis for recurrence rate.

The 5-year overall survival, 5-year disease-free survival, and recurrence rates were included to evaluate long-term oncological outcomes. The results revealed no difference in 5-year overall survival (*p* value = 0.22; 95% CI 0.90 to 1.02; I^2^ = 0%) or recurrence rate (*p* value = 0.08; 95% CI 0.47 to 1.04; I^2^ = 47%), and the 5-year disease-free survival (*p* value = 0.01; 95% CI 1.11 to 2.57; I^2^ = 23%) was slightly better in RATS than in VATS (shown in **Figure 5**).

### Publication Bias

**Figure 6:**
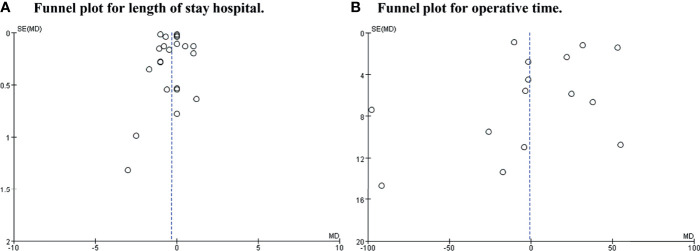
Funnel plot for length of hospital stay and operative time. **(A)** Was length of stay hospital. **(B)** Was operative time.

A funnel plot for length of hospital stay and operative time was drawn to investigate the potential publication bias. The funnel plot shows obvious publication bias between studies (shown in **Figure 6**).

## Discussion

Lobectomy with lymphadenectomy is regarded as the standard surgical treatment for patients with early-stage NSCLC. Due to the popularity of high-resolution CT for screening NSCLC, numerous small early-stage NSCLC (tumor diameter ≤2 cm) are captured (39). Recent reports have shown that sublobar resection or segmentectomy with lymph node sampling has similar outcomes compared with lobectomy for small early-stage NSCLC patients (39, 40). During the past decade, there is sufficient evidence that minimally invasive surgery (MIS) can be used as an alternative standard for NSCLC (5). MIS, including VATS and RATS, could strongly improve short-term outcomes and maintain equivalent long-term outcomes compared with traditional thoracotomy (7, 41). Currently, RATS has been increasingly used worldwide as a new surgical approach for NSCLC.

Although some meta-analyses have demonstrated that RATS is a feasible and safe alternative compared with VATS (7, 8, 42), the debate about the type of operation chosen for NSCLC continues to maintain people’s attention. We read two meta-analyses with great interest published recently by Wu et al. and Ma et al., who aimed to compare the short-term and long-term efficacy between RATS and VATS for NSCLC. Although the research by them was well conducted, unfortunately, we found some flaws in their studies. In the study by Ma et al. (8), we found that the included original articles contained benign lung diseases and metastatic lung cancer, and not all pathologies were NSCLC, which could lead to potential bias in the results. In another study by Wu et al. (7), one study was retracted because this research was fabricated and was included in the analysis of postoperative complications, which could lead to potential flaws in the research. Meanwhile, several new studies were published recently; 26 studies, including 2 randomized controlled trials, 8 propensity score matching studies, and other retrospective studies, were included based on strict inclusion criteria, and an updated meta-analysis was conducted to explore and compare the clinical efficacy of RATS and VATS for patients with NSCLC from 2011 to 2021. Our study included a total of 45,733 patients from 26 studies comparing RATS and VATS, and we focused on both the short-term and long-term oncological outcomes of RATS and VATS for NSCLC.

In short, this meta-analysis did not detect any statistically significant differences in operative time, any complications, tumor size, chest drain duration, R0 resection rate, lymph station, 5-year overall survival, or recurrence rate. However, less blood loss (*p* value = 0.003; 95% CI -65.99 to -14.08), a lower conversion rate to open (*p* value = 0.004; 95% CI 0.45 to 0.85), a shorter length of hospital stay (*p* value = 0.02; 95% CI -0.60 to -0.04; I^2^ = 99%), more lymph node dissection, and a better 5-year disease-free survival (p value = 0.01; 95% CI 1.11 to 2.57; I^2^ = 23%) were captured in RATS than VATS. The results of the meta-analysis revealed that the operative time, any complications, chest drain duration, and number of lymph stations were similar between the two groups, which was consistent with the results reported by Liang et al. (42). Regarding less blood loss and a lower conversion rate to open, the possible reason is that RATS has better visualization and reduces natural tremors. Thorough oncological surgical margins and lymph node dissections are two important malignancy prognosis factors in the surgical approach for NSCLC. There was no significant difference in terms of the R0 resection rate or recurrence rate between RATS and VATS, which showed that both RATS and VATS are feasible surgical techniques for NSCLC. However, more lymph node dissection and a better 5-year disease-free survival were captured in RATS compared with VATS, which might be related to the robot system being flexible operating instruments and having a greater advantage in obtaining lymph nodes, and more lymph node dissection could lead to a better 5-year DFS for patients with NSCLC. Moreover, the 5-year overall survival was similar in the two groups, while a better 5-year disease-free survival was found in RATS, which was similar to the results of a previous study (7).

To evaluate the safety and efficiency of RATS for NSCLC, this meta-analysis included 26 studies and showed that RATS was comparable to VATS. However, this review has some limitations that should be considered. First, there were only two RCTs out of all 26 included studies, which may have contributed to selection bias. Furthermore, of the 26 included studies, the long-term oncological outcomes with NSCLC have not been reported in most of the studies (including the two RCTs that did not report the long-term survival outcomes of NSCLC). Moreover, there was obvious publication bias with the included studies. Therefore, further studies, particularly large-scale prospective studies and RCTs, are expected to assess the effectiveness and safety of RATS for patients with NSCLC.

In conclusion, this systematic review and meta-analysis suggests that RATS is a technically and oncologically safe and feasible approach for NSCLC patients. Large randomized and controlled prospective studies are needed to confirm the superiority of RATS.

## Author Contributions

JZ, QF, YH, and FL designed the project. JZ, QF, and YH extracted the data, made the figures and tables, and wrote the manuscript. LO and FL revised the manuscript. All authors reviewed manuscript. All authors contributed to the article and approved the submitted version.

## Funding

This work was supported by grants from the National Natural Science Foundation of China (No. 82060390).

## Conflict of Interest

The authors declare that the research was conducted in the absence of any commercial or financial relationships that could be construed as a potential conflict of interest.

## Publisher’s Note

All claims expressed in this article are solely those of the authors and do not necessarily represent those of their affiliated organizations, or those of the publisher, the editors and the reviewers. Any product that may be evaluated in this article, or claim that may be made by its manufacturer, is not guaranteed or endorsed by the publisher.
